# Dysregulated TGF-β Production Underlies the Age-Related Vulnerability to Chikungunya Virus

**DOI:** 10.1371/journal.ppat.1005891

**Published:** 2016-10-13

**Authors:** Jennifer L. Uhrlaub, Vesna Pulko, Victor R. DeFilippis, Rebecca Broeckel, Daniel N. Streblow, Gary D. Coleman, Byung S. Park, John F. Lindo, Ivan Vickers, Joshua J. Anzinger, Janko Nikolich-Žugich

**Affiliations:** 1 Department of Immunobiology and the Arizona Center on Aging, University of Arizona College of Medicine, Tucson, Arizona, United States of America; 2 Vaccine and Gene Therapy Institute, Oregon Health and Science University, Beaverton, Oregon, United States of America; 3 Charles River, Ashland, Ohio, United States of America; 4 Division of Biostatistics, Department of Public Health and Preventive Medicine, Oregon Health and Science University, Portland, Oregon, United States of America; 5 Department of Microbiology, University of the West Indies, Mona, Kingston, Jamaica; University of Texas Medical Branch, UNITED STATES

## Abstract

Chikungunya virus (CHIKV) is a re-emerging global pathogen with pandemic potential, which causes fever, rash and debilitating arthralgia. Older adults over 65 years are particularly susceptible to severe and chronic CHIKV disease (CHIKVD), accounting for >90% of all CHIKV-related deaths. There are currently no approved vaccines or antiviral treatments available to limit chronic CHIKVD. Here we show that in old mice excessive, dysregulated TGFβ production during acute infection leads to a reduced immune response and subsequent chronic disease. Humans suffering from CHIKV infection also exhibited high TGFβ levels and a pronounced age-related defect in neutralizing anti-CHIKV antibody production. In vivo reduction of TGFβ levels minimized acute joint swelling, restored neutralizing antibody production and diminished chronic joint pathology in old mice. This study identifies increased and dysregulated TGFβ secretion as one key mechanism contributing to the age-related loss of protective anti-CHIKV-immunity leading to chronic CHIKVD.

## Author Summary

Chikungunya virus (CHIKV) causes an acute febrile syndrome and severe acute and chronic joint disease that, for unknown reasons, especially affects older adults. CHIKV has recently reached the Americas and is threatening the Southeastern USA. At the present there is no approved vaccine or antiviral treatments against CHIKV and treatment is limited to non-steroidal anti-inflammatory drugs (NSAIDs). We have shown here in a mouse model that increased production of the immunosuppressive cytokine TGFβ contributes to the excess of CHIKV disease severity with aging. Blocking of this cytokine reduced early swelling and late joint/foot pathology, and increased production of neutralizing antibodies. Since our data demonstrate high levels of TGFβ in humans experiencing acute CHIKV infection and decreased anti-CHIKV neutralizing antibodies in older CHIKV-exposed humans, these results could pave the way to new treatments against CHIKV disease.

## Introduction

Chikungunya virus (CHIKV) is a re-emerging mosquito-borne alphavirus endemic to West Africa, with outbreaks in many Asian and African countries [1], that causes a febrile illness characterized by rash and arthralgia that is often debilitating [2,3]. The distinctive severity of the joint pain causes those suffering from this virus to assume a twisted protective position, which gave chikungunya disease (CHIKVD) its name meaning “that which becomes contorted” [4]. Although some patients resolve joint pain and swelling within 10–12 days, in up to half of patients symptoms become chronic and can persist for years [5,6].

High viremia during early CHIKVD (10^9−12^ virus copies/mL) enables transmission from person to person via mosquitoes [7]. Since its discovery, CHIKV has widened its geographic range [8], reaching the Caribbean and South America by 2013, with >1M clinical and 25,000 laboratory confirmed cases [9]. The *Aedes* (*Ae*.) species of mosquitoes that carry CHIKV reside in the U.S. (*Ae*. *albopictus* and *Ae*. *aegypti*), and Florida reported autochthonous transmission in 2014 (www.paho.org), highlighting the risk for the U.S., with its CHIKV-naïve and rapidly aging population, to become an epidemic location.

Combined data from human outbreaks and animal models have begun to reveal the pathogenesis of, and immunity against, CHIKV infection. There is a positive association between high levels of serum pro-inflammatory cytokines and CHIKV clearance in humans, monkeys, and mice [10–15]. Type I IFN (α/β) was specifically identified as a key mediator associated with CHIKV clearance [15,16], and expression of the Type I IFN receptor (IFNAR) on non-hematopoietic cells was required for survival of CHIKV [17]. Synovial macrophages are a reservoir for virus and could be involved in early joint swelling [18–20] and subsequent regulation of inflammation. CD4^+^ T cells appear required for the early joint swelling in mouse models [21], whereas their role and the role of CD8^+^ T cells in viral clearance remains controversial [21,22]. B cells infiltrate the footpads and passive transfer of antibodies (Ab) can control infection in mice [22–24]. Humans also display a robust anti-CHIKV Ab response that is believed to be protective [18,20]. However, the exact contribution of each facet of the immune response to disease presentation and resolution remains incomplete. It remains unclear (i) how the innate response coordinates the adaptive response during CHIKV infection; (ii) what the impact of cytokine, T cell, and B cell responses may be on disease severity and length; and (iii) how that may be altered in the context of specific risk factors such as advanced age. Indeed, the greater risk of persistent and severe CHIKV disease among the elderly is likely due to one or more aging-related defects in the innate and adaptive immune response.

To elucidate such potential defects, we developed a mouse model which recapitulates age-related clinical outcomes observed in CHIKV-infected elderly humans. We report that increased production of TGFβ is linked to qualitative and quantitative impairments in B and T cell responses, which fail to clear the virus. We show that anti-TGFβ Ab treatment can prevent age-related increases in CHIKV disease severity, reduce joint pathology, and improve production of neutralizing Ab. Given that TGFβ is also elevated in humans suffering from CHIKVD, we propose this pathway as a possible target in treating CHIKV infection in older adults.

## Results

### Age-related increases in acute CHIKV-induced joint swelling

**Fig 1:**
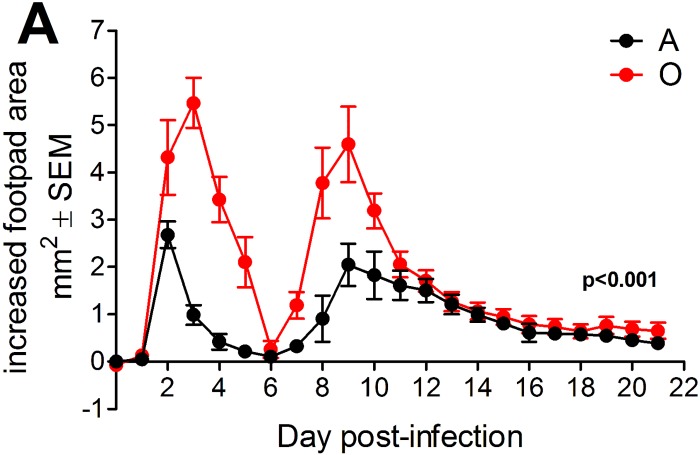
Age increases acute CHIKV-induced joint swelling. (A) A (12 weeks) and O (18–20 months) B6 mice were inoculated via f.p. and swelling was measured daily as described in Methods. Data are mean ±SEM (*n* = 10–16 per group). Statistical significance was determined using mixed model, repeated measures analyses of variance (ANOVA) as detailed in Statistics. ***P< 0.001.

To define age-related changes in anti-CHIKV immunity, we infected C57BL/6 (B6) mice with CHIKV strain SL15649. Footpad (f.p.) CHIKV inoculation in adult mice results in early biphasic foot swelling, peaking on day 3 and 8–9 and corresponding to an early, innate, and a later, adaptive, phase of the response, and resolving by d16 [21,24]. We confirmed these results (Fig 1) and extended them to old mice. Importantly, old (O) mice exhibited significantly increased swelling as compared to adult (A) during both phases of the immune response (Fig 1). In addition, in O mice peak swelling in both phases was sustained longer and at higher levels than in A animals (Fig 1). However, the onset and resolution of swelling occurred in both groups by day 16 post-infection (p.i.; Fig 1). We did not observe swelling in the non-injected or saline-injected contralateral foot and found no CHIKV-specific mortality in either age group

### Old mice exhibit delayed and incomplete CHIKV clearance

**Fig 2:**
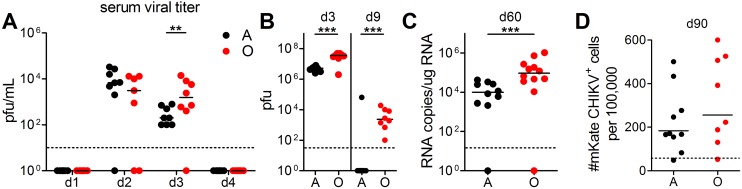
Prolonged CHIKV viremia with age and evidence of persistent infection. (A) Serum and (B) CHIKV-inoculated feet were harvested on indicated days post-infection and assayed for viral titer by plaque assay. (A) Serum viral titers on days 1–4 post-infection and (B) viral titer of CHIKV feet on days 3 and 9 post-infection (*n* = 7–8 per group). (C) Genome copies of virus in CHIKV feet on day 60 post-infection (*n* = 16–18 per group). (D) CHIKV-inoculated feet were harvested at day 90 p.i. and assayed for the presence of fluorescent infectious virus by co-culture on C6/36 insect cells (n = 8–10 per group). Dashed line indicates limit of detection for all assays. Horizontal lines indicate the median. Statistical significance determined on log-transformed data by unpaired student’s *t*-test. *P< 0.05; **P< 0.01; ***P< 0.001.

To examine whether increased foot swelling in O CHIKV-infected mice correlates to increased viremia, we measured viral titers in the serum, inoculated foot (Fig 2), and non-inoculated foot (S1 Fig) by plaque assay. In the blood, CHIKV was first detected on d2 p.i., (Fig 2A). By d3 p.i., O mice exhibited significantly higher CHIKV titers, suggestive of delayed viral control, but by d4, both O and A mice resolved viremia (Fig 2A), similar to findings in other viral [25]and bacterial [26] models where O mice usually manage to control systemic virus following a delay, relative to A mice. Support for the idea that O mice exhibit delayed viral control was even more remarkably illustrated by data from inoculated feet, where O mice displayed 10-fold higher viral loads than in A on d3 p.i. (Fig 2B). Despite the absence of joint swelling, CHIKV was also detectable on d3 in the contralateral, non-inoculated foot at ~1000 fold lower levels compared to the inoculated foot (S1 Fig). By d9 p.i., infectious CHIKV dropped below the limit of detection in the feet of most A mice but remained detectable in both inoculated (Fig 2B) and contralateral (S1A Fig) feet of the O animals. Viral genomes could be detected in the inoculated footpads on d60 p.i., with significantly higher viral genome copies in the O mice (Fig 2C). These results demonstrate impaired virus control with aging, consistent with data from Rhesus macaques [15]. Finally, very low levels of fluorescent infectious virus were recovered from both A and O mice at 90 days post-infection (Figs 2D and S1B) demonstrating for the first time in an animal model that replicating CHIKV persists far beyond the acute phase in the infected joints. This finding is consistent with evidence for replicating virus isolated from a single patient experiencing chronic CHIKVD [18]. While O and A mice did not show statistically significant difference in infectious viral load on d90 at this experimental power, there was a trend of higher levels in O mice, which will have to be substantiated in future experiments. While delayed viral control in O mice could suggest a link with increased early swelling, viral load did not directly correlate with, and is probably not the sole determining factor for, swelling. This follows from data showing that marked swelling was present at times where infectious viral titers were below the limit of detection for plaque assay, as is the case with most adult animals on d9 p.i. and with both age groups during chronic CHIKVD. This relationship between infectious virus, swelling and joint pathology as a function of age will, therefore, require further investigation, but data so far are consistent with prior literature suggesting the presence of host response components.

### Lymph node cellularity and cellular immune responses against CHIKV are reduced in old mice

**Fig 3:**
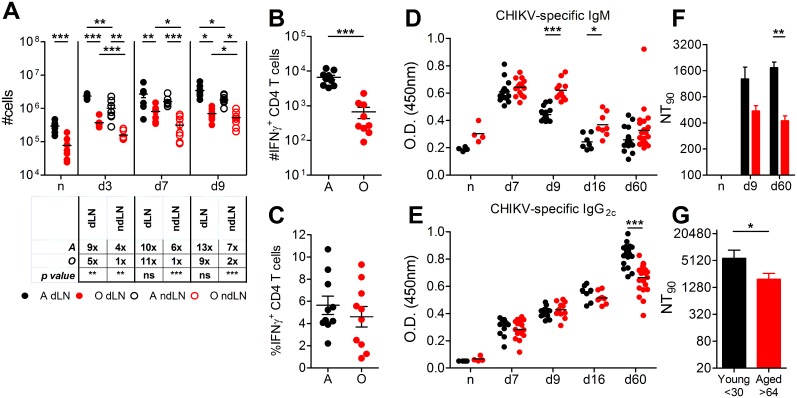
Age-related impairment of adaptive immune response to CHIKV. (A) Popliteal LNs collected and quantified from either naïve or CHIKV-infected A and O mice at day 3, 7, or 9 post-infection. The LN draining from the CHIKV-inoculated foot is indicated as dLN and from the non-inoculated foot as ndLN. Table under graph indicates the average fold-increase from naïve for each age in either the dLN or ndLN (*n* = 6–8 per group). Horizontal lines indicate the median. Statistical significance determined by student’s *t*-test. (B-C) Lymphocytes from popliteal LNs on d7 post-infection were stimulated with CHIKV peptides in the presence of protein transport inhibitor. Total number of IFNγ^+^ CD4 T cells (B) and frequency (C) for each age. Data are mean ± SEM (*n* = 10 per group). Statistical significance determined by unpaired Student’s *t*-test. (D) CHIKV-specific IgM and (E) IgG2c in serum determined by ELISA at the indicated day post-infection. Data are mean (*n* = 4–24 per group). Statistical significance was determined by two-way ANOVA with Bonferroni post-test. (F) Plaque reduction neutralizing test on serum from days 9 and 60 post-infection. Data are mean + SEM (*n* = 12 per group). Statistical significance was determined by two-way ANOVA with Bonferroni post-test. (G) Serum samples collected from patients experiencing acute CHIKV-disease were evaluated by plaque reduction neutralizing test. Data are mean + SEM (*n* = 24 young and 15 aged). Statistical significance was evaluated by unpaired student’s *t*-test. In all panels black indicates A, red indicates O; * *P*< 0.05; ** *P*< 0.01; *** *P*< 0.001.

Increased foot swelling in O mice could be caused by direct cytopathic virus effects, by immunopathological actions of innate or adaptive cells or molecules (cytokines), or by a combination of both. To discern between these possibilities we analyzed maintenance and recruitment of various cells into the lymph nodes (LN) of O mice. LN from naïve O mice were visibly smaller than those in A mice (S2A Fig). Further, despite an increase in the size of the draining LN (dLN) and non-draining LN (ndLN) on d3 of CHIKV infection, O LN never reached the size of A LN at any time point (S2A Fig). Total cellularity of naïve and d3, 7, and 9 p.i. dLN (Fig 3A) was also significantly lower in O at all time points compared to A LN. This suggests an inability of the O LN to expand, recruit and/or maintain a sufficient number of cells to make up for the deficit in naïve LN, consistent with recent data [27]. The reduced LN reaction was evident in the ndLN in O mice as well, which exhibited only minimal, if any increase in total cell numbers (Fig 3A) despite the local presence of the infectious virus at these times (S1B Fig). An analysis of natural killer (NK), dendritic cells (DC) and macrophages revealed that these cells were either reduced from the beginning and/or failed to accumulate to the same levels as in A animals (S2B Fig), suggesting that none of them would be likely to account for excess foot swelling in O mice. We also found reduced CD4^+^, CD8^+^ and B cell numbers in the LN of naïve O mice and none of these populations were able to expand to equivalent numbers found in LN of A mice (S2C Fig) following CHIKV infection.

To precisely evaluate the T cell responses, we identified dominant I-A^b^ restricted CHIKV regions (S2D Fig), with the E2_2805-2820_ epitope being absolutely immunodominant (S2E Fig). We found a tenfold reduction in absolute numbers of IFNγ^+^ CHIKV-specific CD4^+^ cells in O mice (Fig 3B), which would not have been revealed by percentage/frequency comparison (Fig 3C, a trend but no significant A to O difference). During our initial screen of peptide pools, we did not find differences in the CD8 IFNγ responses with aging in the spleen (S2H Fig), and subsequent preliminary analysis failed to discover differences in the LN, although cellularity of LN was sharply reduced (Fig 2). We conclude that numerically, both CD8 and CD4 responses were reduced, and, at face value, this reduction is inconsistent with the idea that these cells could mediate enhanced immunopathology in O mice.

### Aged mice and humans generate poorly neutralizing CHIKV-antibodies

Reduced CD4^+^ T cell responses in O mice, together with reduced B cell numbers in the dLN, could lead to impaired humoral responses in O mice. We found that both A and O mice produced anti-CHIKV IgM antibody by d7 p.i., yet, while IgM levels dropped in A mice, they remained significantly higher in O mice over adult on d 9 and 16 post-infection (Fig 3D), consistent with reduced efficacy in class switching in O mice [28,29]. Amounts of anti-CHIKV IgG_2c_ Ab, the isotype considered to be most protective against CHIKV [30], did not increase to the same levels in O mice compared to A counterparts on d16 and 60, and the difference was significant at d60 (Fig 3E), following the trend of the total IgG Ab (S2F Fig), suggesting an impaired memory Ab response. An exception was anti-CHIKV IgG2b, that trended higher in O mice on d9 and was significantly elevated by d16 (S2G Fig). The IgG2b isotype is associated with a “suppressive” cytokine environment that includes production of TGFβ [31].

We next tested the neutralizing capacity of A and O serum in a plaque-reduction neutralization test (PRNT). We found that the neutralizing potency of O serum was trending lower than in A mice on d9, and that difference was statistically significant on d60 (Fig 3F). This also held true for CHIKV-infected humans, where serum from people >65y contained significantly lower neutralizing Ab titers than in those <30y (Fig 3G). Therefore, decreased amounts, suboptimal iso/allotype and reduced neutralizing potency of Ab with age all likely contribute to the increased disease severity, incomplete viral control and elevated incidence of chronic disease in the elderly.

### Age-related dysbalance in pro- and anti-inflammatory mediators during CHIKV infection

**Fig 4:**
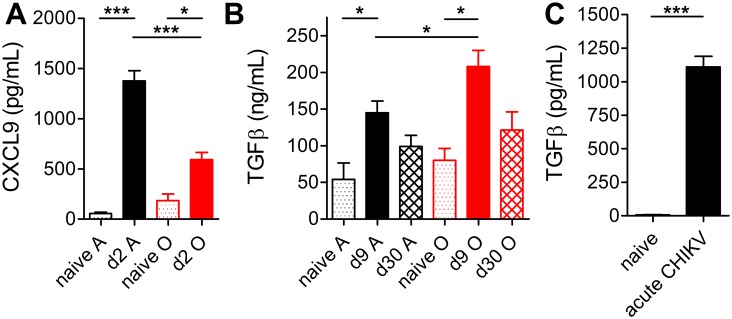
Dysregulated cytokine production with age. Serum was collected from A and O mice and assayed by ELISA for (A) CXCL9 or (B) TGFβ concentration at days 2 or 9 and 30, respectively. Data are mean + SEM (*n* = 3 naïve and 7–8 infected per age). (C) Human samples from IgM-positive CHIKV patients or age and sex-matched controls were assayed for Free-active TGFβ cytokine by ELISA. Data are mean + SEM (*n* = 39 each group). Statistical significance was evaluated by unpaired student’s *t*-test. * *P*< 0.05; ** *P*< 0.01; *** *P*< 0.001.

The above age-related defects in the CD4^+^ T cell and the humoral responses prompted us to evaluate serum cytokine and chemokine profiles in A and O mice following CHIKV infection. While most of the cytokines and chemokines assayed by Luminex array exhibited no significant age-related differences, or exhibited differences that could not be validated by ELISA (e.g. differences in IL-10, S3 Fig), we found an early (d2 p.i.) and significant under-induction of CXCL9 in O mice, which was confirmed by ELISA (Fig 4A). CXCL9 is a proinflammatory chemokine that functions as a chemoattractant for activated lymphocytes, and its lower production could have contributed to delayed and incomplete recruitment to the dLN, an issue currently under investigation.

Moreover, O mice exhibited a significantly greater increase in TGFβ on d3 (S4A Fig) and d9 p.i. relative to A counterparts (Fig 4B), although by d30 these levels returned to baseline in both A and O mice (Fig 4B). TGFβ is a pleiotropic cytokine with diverse effects on the immune system that are incompletely understood. TGFβ operates as a switch-factor for murine antibody isotypes, inducing IgG2b, as well as for mediation of leukocyte recruitment and activation [32,33]. The increased levels of TGFβ-switched IgG2b anti-CHIKV Ab on d16 post-infection of O mice (S3G Fig) led us to hypothesize that anti-CHIKV immunity in O mice is improperly coordinated and that excessive production of TGFβ contributes to increased CHIKVD in O mice. We also found very high levels of free-active TGFβ in sera of acute CHIKV patients (Fig 4C), which validated TGFβ as a potentially relevant cytokine in CHIKVD in both humans and mice.

### TGFβ blockade restores Ab responses and prevents age-increased CHIKVD

**Fig 5:**
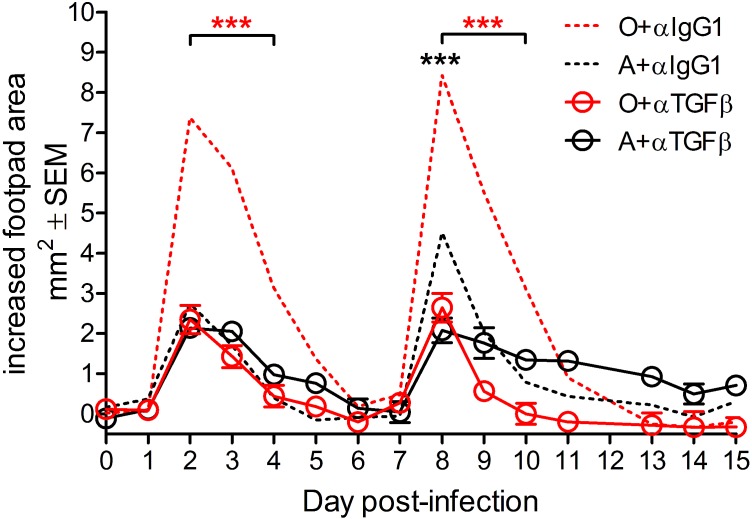
Blocking TGFβ prevents acute CHIKV-induced disease in O mice. A and O B6 mice were inoculated and treated with 100ug of anti-TGFβ antibody or isotype control and swelling was measured daily as described in Methods. Data are mean ± SEM (n = 8 per group). Statistical significance was determined using mixed model, repeated measures analyses of variance (ANOVA) as detailed in Statistics. Red stars indicate reduction of swelling in O mice from αIgG1 to αTGFβ treated; black stars indicate reduction of swelling in A mice from αIgG1 to αTGFβ treated.

To test the above hypothesis, we treated A and O mice with footpad injections of anti-TGFβ Ab or isotype control on d-1, 1, 3 and 5 p.i. and demonstrated that this treatment reduced the concentration of TGFβ in serum in O mice close to or to the levels of TGFβ in A mice (S4A Fig). Reducing the serum of concentration of TGFβ did not have a direct effect on CXCL9 concentration (S4B Fig) suggesting that in O mice systemic CXCL9 may not be depressed due to elevated TGFβ. A somewhat more complex situation was seen in the case of Type I Interferon (S4C Fig), known to be required for control of early CHIKV infection [16]. O mice produce significantly less Type I Interferon than A (S4C Fig) on d2 p.i., consistent with results in old non-human primates [15]. However, that difference disappeared under TGFβ blockade, both because TGFβ blockade slightly reduced production of Type I IFN in A mice and slightly increased its production in O mice (S4C Fig). Importantly, TGFβ blockade did effectively reduce both peaks of acute foot swelling in O mice (red dashed vs. red solid line) to the levels observed in A mice (Fig 5), strongly suggesting that high levels of TGFβ contribute decisively to the age-related increase in acute CHIKVD. TGFβ Ab treatment in CHIKV infected A mice did not reduce swelling during the early, but did during the late acute phase (Fig 5, d8, black dashed vs. black solid line). It should be noted that TGFβ neutralization did not prevent swelling altogether, which could be due to the fact that systemically TGFβ was not completely neutralized in O and was only marginally reduced in A mice (S4A Fig) or could suggest the existence of other, age-independent, mediators of acute CHIKVD. Experiments assessing local levels of different cytokines, including TGFβ and Type I IFN, are in progress to further resolve this issue.

Further, TGFβ reduction/blockade did not exert a direct anti-viral effect, as determined by measuring viral titers in serum on days 1–4 (S5A Fig) and tissues on days 3 and 9 (S5B and S5C Fig), where groups with blockade did not have appreciably lower viral titers compared to control groups. TGFβ reduction/blockade also did not promote full clearance of viral genomes from the tissue (S5D Fig). This data taken together suggests that CHIKV persistence is driven by elevated TGFβ, but likely also by other factors, most notably host defense mechanisms. Genetic ablation of TGFβ signaling in specific cell subsets will be necessary to conclusively discriminate between these possibilities.

**Fig 6:**
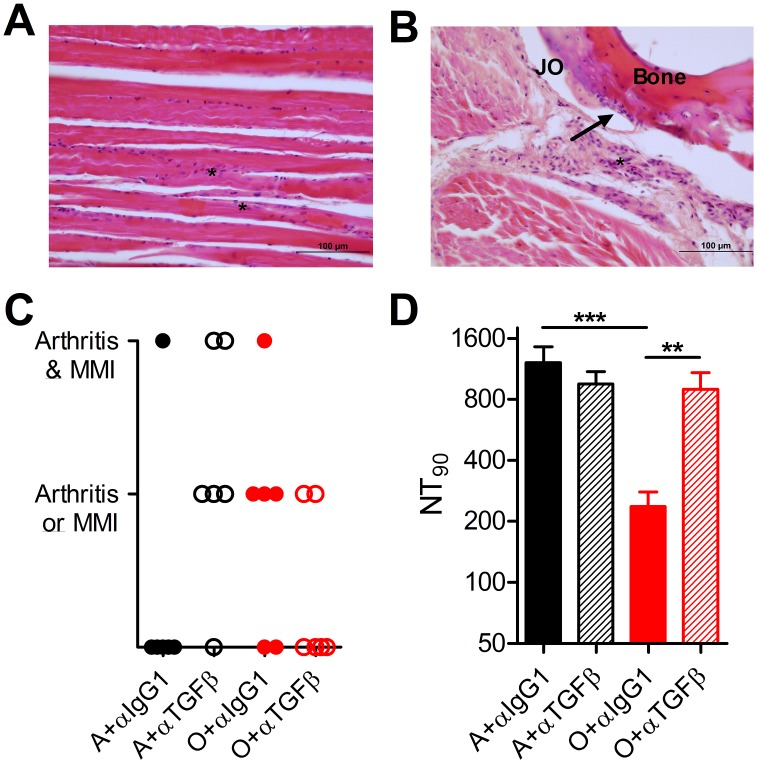
Blocking TGFβ prevents chronic CHIKV-induced disease and restores neutralizing antibody titers in old mice. Blinded H&E stained histology sections were evaluated by an anatomic pathologist for arthritis and metatarsal muscle Inflammation (MMI). (A) Representative metatarsal muscle inflammation. Astrisks indicate areas of inflammatory cells, muscle cell degeneration and pallor. H&E 40X magnification, bar is 100uM. (B) Representative arthritis. Arrow indicates area of articular cartilage erosion with infiltrate by neutrophils. Asterisk is an area of inflammation of the joint capsule characterized by the infiltration of neutrophils and mild edema. JO = Joint. H&E 20X magnification. (C) Mice were scored as having either metatarsal muscle inflammation (MMI), arthritis, or both. (D) Plaque reduction neutralizing antibody titers were determined at day 90 post-infection. Data are mean ± SEM (n = 11–12 per group). Statistical significance evaluated by unpaired student’s *t*-test * *P*< 0.05; ** *P*< 0.01; *** *P*< 0.001.

TGFβ reduction/blockade during acute infection also reduced the incidence of chronic arthritis and restored neutralizing Ab responses against CHIKV in old mice (Fig 6). When hematoxylin/eosin (H&E) stained tissue sections were evaluated for synovitis, arthritis, and metatarsal muscle inflammation on d90 p.i. using a previously described scoring system [22], we found that control-treated O mice exhibited increased frequency of chronic arthritis and/or metatarsal muscle inflammation (4 of 6 mice) as compared to A (1 of 6 mice, *P* = 0.07, chi-square test) (Fig 6A and 6B). Importantly, that incidence was reduced by 50% when TGFβ was blocked during acute infection in O mice (Fig 5C), whereas, neutralization of TGFβ in A mice made the late joint pathology worse (Fig 5C), suggesting that in a properly controlled response in A mice, TGFβ plays a protective role in joint infiltration and pathology.

Finally, we evaluated the neutralizing Ab capacity on d90 p.i. and found that anti-TGFβ blockade during acute infection restored CHIKV-neutralizing Ab titers to high, adult-like levels in O mice, but did not affect neutralizing Ab responses in A mice (Fig 5D). Taken together, our results identify increased, dysregulated TGFβ secretion during very early, acute infection as a key, specific mechanism contributing to the age-related loss of immune system function and increased joint pathology in the course of CHIKV infection.

## Discussion

CHIKV is an emerging disease with pandemic potential and pronounced acute and prolonged disability, particularly in older adults. A mouse model of A and O infection using footpad inoculation of B6 mice, described in this report, provides important clues on the basis of age-related vulnerability to CHIKVD. The disease in O animals was marked by enhanced prolonged viremia, more severe early swelling and late footpad joint and connective tissue pathology. We also present evidence that live, replication competent, CHIKV persists in the tissues of both A and O mice. This suggests that increased chronic CHIKVD with age is not due to differential viral persistence but is rather a consequence of how persistence is controlled. Importantly, we show that aged animals generated a quantitatively and qualitatively defective immune response at both innate and adaptive levels. We demonstrate that dysregulated TGFβ cytokine secretion decisively contributed to both enhanced CHIKVD and to defects in protective immunity. We further report that this dysregulation is age-specific and does not play a role in young mice. This is supported by the fact that neutralization of TGFβ in A animals did not erode their B cell response and may have been somewhat detrimental to the late joint pathology. Finally, similar signs of immune dysregulation with CHIKVD were observed in humans, including elevated TGFβ in adult and older humans and reduced neutralizing Ab titers in older humans. Our human studies were not powered or designed to conclusively assess whether in humans there are any age-related differences in TGFβ production, an issue that will have to await further studies. Also of note, we saw no sex differences in TGFβ production between male and female human subjects, and in one experiment with limited numbers of female old mice, we saw the same excess swelling and reduced neutralizing Ab responses as seen in old males However, this study chiefly studied male mice, and therefore sex differences in the susceptibility to CHIKVD with age remain to be explored.

Based on this, we propose that treating the age-related changes in the immune system (and, likely, in other systems and organs) as a simple continuum of processes known to operate in younger age could be conceptually limiting, and even erroneous in some situations. Our results are at least in part consistent with an altered state of the old immune system, where some of the rules that operate in youth no longer apply, due to dysregulated homeostasis. Results of recent studies on the maintenance of the naïve T cell pool and its diversity by us [34–36] and others [37–39] are consistent with that idea, and may suggest re-thinking of the conceptual framework within which we consider age-related changes in function.

Generation of an effective immune response requires coordinated activation of early innate and late adaptive immune responses, and any age-related changes in either of the two arms could profoundly affect the ability to fight off the virus initially or control persistent infection. With aging we found reduced levels of CXCL9, a chemo-attractant mostly secreted by macrophages, and responsible for the recruitment of lymphocytes during viral infections [40,41] which could explain the reduced numbers of lymphocytes recruited to dLN and/or infected joints. Yet that was not the only issue found in the O LN, which were smaller in size even before infection, and never reached the degree of size or cellularity measured in the A counterparts. There could be multiple reasons for this, including a well-described decline in naïve CD8^+^ [36,37] and CD4^+^ [39] numbers, and the more recently described age-related degradation of LN stromal architecture, which may render it incapable of supporting the youthful number and diversity of cell types. Indeed, recent data points to the changes in stromal architecture of the LN with age in the steady state [42] and the inability of LN to recruit and properly direct migration of lymphocytes following infection [27] where the above mentioned defect in CXCL9 production could play a role.

In addition, immunodominant CD4^+^ T cell epitope identification allowed us to reveal a reduced anti-CHIKV IFNγ response of old CD4^+^ T cells. This provides further evidence suggesting that prolonged clinical pathology observed at the site of viral infection in O mice is probably not mediated by CHIKV-specific Th1 CD4^+^ or CD8^+^ T cells, as their numbers and function are decreased in aging. Moreover, we did not observe Th17 or increased Treg cells in CHIKV infection so far Consistent with depressed Th1 immunity, we found similar initial viral titers but an age-related delay in CHIKV control in O mice. These observations closely parallel the results obtained in CD4-/- and IFNγ-/- mice [30], where defects in both cellular (CD4^+^ cells) and humoral (Ab) immunity contribute to impaired immunity to CHIKV. We therefore conclude that the CD4^+^ and B cell lymphopenia measured in the LN, decreased Th1 response of CHIKV-specific CD4^+^ T cells and the action of TGFβ likely contribute to the total IgG and IgG2c antibody deficiency and to reduced CHIKV-neutralizing titers on day 60 post-infection.

Of importance, the higher TGFβ levels in the O mice led not only to increased early conversion into IgG2b isotype but also established an environment conducive to swelling and tissue pathogenesis. Increased production of TGFβ in O mice during acute infection is not unique to CHIKV as it was found in the West Nile Virus infection (S6 Fig) and following *Encephalitozoon cuniculi* infection of old mice [43]. However, since TGFβ blockade reversed all the above phenomena in CHIKV infection, our data suggest that with aging, increased TGFβ levels likely tipped the balance away from generation of an efficient and protective immune response and towards chronic arthritis. While many details remain to be elucidated about the exact mechanistic functioning of the TGFβ axis in old mice undergoing CHIKV infection and CHIKVD, our results reported herein identify TGFβ as one key mechanism behind age-related vulnerability to CHIKVD. Further, our studies point to this cytokine and its signaling pathway as a potential target for immune intervention to remedy the pathology associated with CHIKV infection, and present preliminary validation of this target in humans.

## Methods

### Ethics statement

Mouse studies were carried out in strict accordance with the recommendations in the Guide for the Care and Use of Laboratory Animals of the National Institutes of Health. Protocols were approved by the Institutional Animal Care and Use Committee at the University of Arizona (IACUC #08–102, PHS Assurance Number: A3248-01). Footpad injections were performed under isoflurane anesthesia. Euthanasia was performed by isoflurane overdose or cervical dislocation. All collection and use of samples from human subjects was approved by the Ethics Committee at The University of the West Indies and the Institutional Review Board at the University of Arizona. Informed consent was written and provided by the subject or their legal guardian.

### Mouse experiments

O (18 months) and A (12 weeks) male C57BL/6 (B6) mice were purchased and/or obtained from the National Institute on Aging Rodent Resource via the Charles River Laboratories (Frederick, MD and Kingston, NY) and/or The Jackson Laboratory (Bar Harbor, ME). Mice anesthetized with isoflurane were infected subcutaneously in the footpad (f.p.) with 1000pfu of CHIKV as previously described [44]. Foot swelling was measured daily with calipers until d21 p.i. Footpad area was determined as (height x width) and expressed as increase over d0. No swelling was observed in the non-injected or saline-injected contralateral foot. All CHIKV experiments were conducted within U.S. Department of Agriculture and CDC-inspected biosafety level 3 facilities at the University of Arizona.

### Virus and titer

CHIKV strain SL15649 (Genbank accession no. GU189061) was isolated from serum of a febrile patient in Sri Lanka in 2006 and was propagated twice in Vero cells before the generation of an infectious cDNA clone, used to previously establish mouse CHIKV infection [44]. The pMH56.2 plasmids encoding SL15649 CHIKV and SL15649 CHIKV expressing mKate were generously provided by Dr. C. E. McGee and Dr. M. T. Heise [45]. Virus titer was determined by plaque assay on Vero cells [44].

### Viral quantification

Infectious viral titers were determined by a standard plaque assay on Vero cells as described in [44]. CHIKV RNA loads were measured using quantitative real time reverse transcription PCR with the following primers and probe: CHIKV-9482F 5′-GGAACGAGCAGCAACCTTTG-3′; CHIKV-9931R, 5′-ATGGTAAGAGTCTCAGACAGTTGCA-3′; and probe CHIKV-9870F, 5′-GGAATAAGGGCTTGT-3′ from viral RNA isolated as previously described [15]. Gene amplicons served as quantification standards (sensitivity, 10 to 100 copies). qRT-PCR was performed and analyzed using ABI StepOne Plus real-time PCR system (Applied Biosystems). Persistent viral infection was determined by culturing tissue lysate on C6/36 insect cells for 3 days followed by FCM analysis to detect infected cells. Samples were acquired using a BD LSR Fortessa cytometer (BD Bioscience, San Jose, CA) and analyzed by FlowJo software (Tree Star, Ashland, OR).

### Flow cytometry and intracellular cytokine staining

Accutase-treated (eBioscience, San Diego, CA) popliteal lymph nodes were disassociated over a 40uM cell strainer. Following Fc block, cells were incubated overnight in a saturating dose of mAb against CD3, CD4, CD8α, CD19, CD11b, CD11c, NK1.1 and F4/80 (eBioscience, San Diego, CA), stained with Live/Dead Yellow (Life Technologies, Grand Island, NY) and analyzed as below. Peptide stimulation was in the presence of protein transport inhibitor (eBioscience, San Diego, CA) as described [25]. Overlapping peptide pools (15mer, overlapping by 5) for the 9 proteins of CHIKV were used to determine the immunodominant regions of E2 and NSP1. Libraries of these regions (21^st^ Century, Marlboro, MA) were used to determine individual immunodominant epitopes. Samples were acquired using a BD LSR Fortessa cytometer (BD Bioscience, San Jose, CA) and analyzed by FlowJo software (Tree Star, Ashland, OR). Cell counts were extrapolated from either a hand count on a hemocytometer or by CBC differential collected on a Hemavet LV (Drew Scientific, Waterbury, CT) instrument. The two counting methods were confirmed to be consistent.

### Antibody quantification, isotyping and neutralizing titers

Ab titers were assessed using a CHIKV infectious cell lysate-based enzyme-linked immunosorbent assay (ELISA). Briefly, CHIKV-infected lysate was generated by infection of primary human fibroblasts and used to coat 96 well Immulon 2 HB plates (Thermo Labsystems, Franklin, MA), with uninfected lysate used as control. Plates were blocked with PBS-0.05% Tween-20 + 5% dry nonfat milk. Serum was diluted 1:50 in the same blocking buffer, incubated for 1h at 22°C, incubated with horseradish peroxidase-labeled goat anti-mouse IgG (KPL, Gaithersburg, MD) or anti-IgM, IgG1, IgG2b or IgG2c (Southern Biotech, Birmingham, AL) and developed with 3,3′,5,5′-Tetramethylbenzidine dihydrochloride (Sigma-Aldrich, St. Louis, MO). Reaction was terminated with 1M H_2_SO_4_ and absorbance measured at 450 nm. Plaque reduction neutralization test assay was done on Vero cells and neutralization titers were determined as the serum dilution with a 90% reduction in plaques (NT_90_) compared to wells infected with CHIKV in the absence of serum.

### Human sample collection and evaluation

Between July 2014 and April 2015, blood samples were submitted to the Department of Microbiology at the University of the West Indies to be tested for the presence of CHIKV IgM antibodies. This resulted from an enhanced fever and rash surveillance initiative by the local Ministry of Health as part of the preparedness and response plan for CHIKV introduction in the Island. Samples were tested using the Anti-CHIKV IgM human ELISA kit (Abcam, Cambridge, MA, USA). The Centers for Disease Control and Prevention (CDC) reported an overall sensitivity and specificity of 88% and 90%, respectively. For these studies, serum samples (n = 24 <30 years and n = 15 >65 years; 100μL volume) from identified IgM^+^ CHIKV patients were tested alongside age- and sex-matched CHIKV-naïve controls from Tucson, Arizona.

### Serum cytokine assays

ELISAs for mouse MIG/CXCL9 (R&D Systems Inc, Minneapolis, MN), TGFβ (eBioscience, San Diego, CA), Free-active TGFβ for humans (BioLegend, San Diego, CA) and the Luminex multiplex mouse cytokine assay (Life Technologies, Inc.) were performed following manufacturer instructions.

### TGFβ blockade

100μg of TGFβ antibody, clone 1D11.16.8 (BioXCell, West Lebanon, NH) or IgG1 isotype control, clone MOPC-21 (BioXCell, West Lebanon, NH) were injected via f.p. route on days -1, 1, 3, and 5 p.i. in 20μl saline.

### Histology

Following euthanasia, foot and ankle tissues were collected and fixed in 10% neutral buffered formalin for 24 hours, then processed and embedded into paraffin blocks. Hematoxylin and Eosin (H&E) stains were performed on 5μ sections of tissue cut from the formalin fixed, paraffin embedded (FFPE) blocks.

### Statistical analysis

Data were analyzed using Prism Graph Pad software and the statistical test referenced in each figure with the exception of Figs 1 and 5. These were analyzed by mixed model, repeated measures analyses of covariance (ANOVA), with group as a between group factor and time (days post infection) as a within group factor and their interaction were used to compare differences in performance among all groups over time for analyzing outcome (increased footpad area). In a typical experiment using repeated measures, two measurements taken at adjacent times are more highly correlated than two measurements taken several time points apart; therefore, we used a first-order autoregressive (AR1) covariance structure to account for within-subject correlation. Due to the small sample size, other more complex covariance structures were not considered. Tukey-Carmer multiple comparisons correction was used to control overall type I error rate.

## Supporting Information

S1 Fig (A) Contralateral, non-inoculated feet were harvested on indicated days post-infection and assayed for viral titer by plaque assay (*n* = 7–8 per group). Horizontal lines indicate the median. (B) FCM plots of CHIKV mKate positive C6/36 insect cells after co-culture with tissue homogenate collect at 90 days post-infection. The left plot is mock infected, the middle is a representative positive from the data shown in Fig 2D, and the right is an example of a mouse (a statistical outlier) with a very high quantity of persistent virus.
(TIF)

S2 Fig (A) Visual disparity between popliteal LNs collected from either naïve or CHIKV-infected A and O mice at day 3 post-infection. The LN draining from the CHIKV-inoculated foot is indicated as dLN and from the non-inoculated foot as ndLN in all panels. (B-C) Absolute number of NK cells, dendritic cells (DCs), macrophages, CD4^+^ T cells, CD8^+^ T cells, and B cells as determined by FCM analysis. Table under graph indicates the average fold-increase from naïve for each age in either the dLN or ndLN (*n* = 6–8 per group). Horizontal lines indicate the median. Statistical significance determined by student’s *t*-test. (D) dLN stimulated with overlapping peptide pools for each region of CHIKV in the presence of protein transport inhibitor to determine frequency of IFNγ^+^ CD4^+^ T cells. (E) dLN stimulated with individual peptides from the E2 region of CHIKV to determine dominant epitope. (F) CHIKV-specific total IgG and (G) IgG2b in serum was determined by ELISA at the indicated day post-infection. Data are mean (*n* = 4–24 per group). (H) Splenocytes stimulated with overlapping peptide pools for each region of CHIKV in the presence of protein transport inhibitor to determine frequency of IFNγ^+^ CD8^+^ T cells. Statistical significance was determined by two-way ANOVA with Bonferroni post-test. *P< 0.05; **P< 0.01; ***P< 0.001.
(TIF)

S3 Fig Cytokine and chemokine changes in the course of the first week of CHIKV infection in A and O mice. All panels: Serum from A and O CHIKV infected mice were assayed by Luminex for indicated cytokine levels. Statistical significance was determined by one-way ANOVA with Bonferroni post-test. *P< 0.05; ***P< 0.001.
(TIF)

S4 Fig TGFβ blockade by antibody treatment is transient and does not influence the production of CXCL9 or Type I Interferon. (A) Local administration of anti-TGFβ antibody reduces serum concentration of TGFβ. Data are mean + SEM (*n* = 6–13 per group). Statistical significance was evaluated by unpaired student’s *t*-test. (B) Serum was collected from A and O mice +/- anti-TGFβ blockade and assayed by ELISA for CXCL9 at day 2 post-infection. Data are mean + SEM (*n* = 10 per group). Statistical significance was evaluated by unpaired student’s *t*-test. *P< 0.05; **P< 0.01; ***P< 0.001. (C) Type I Interferon was evaluated by bioassay (Vesicular Stomatitis Virus) on days 1–4 of CHIKV infection [46]. Data are mean +/- SEM (*n* = 5–15 per group). Statistical significance was determined by two-way ANOVA with Bonferroni post-test. ***P< 0.001.
(TIF)

S5 Fig Viral control in O mice is not altered by TGFβ blockade. Serum (A) and CHIKV-inoculated feet (B-D) were evaluated on day 3 (B) and 9 (C) post-infection for viral titer by plaque assay. (*n* = 5–8 per group). (D) Genome copies of virus in CHIKV feet on day 60 post-infection (*n* = 16–18 per group).
(TIF)

S6 Fig TGFβ is produced in O mice during acute West Nile Virus (WNV) infection. Serum was collected from A and O mice and assayed by ELISA for TGFβ concentration at day 10 post-infection. Data are mean + SEM (*n* = 7–8 naïve and 7–10 infected per age). Statistical significance was evaluated by unpaired student’s *t*-test. *P< 0.05.
(TIF)
