# Supplementary figures and images for: Dysregulated TGF-β Production Underlies the Age-Related Vulnerability to Chikungunya Virus
Source: PLoS Pathog. 2016 Oct 13;12(10):e1005891. doi: 10.1371/journal.ppat.1005891 (PMC5063327; doi:10.1371/journal.ppat.1005891)

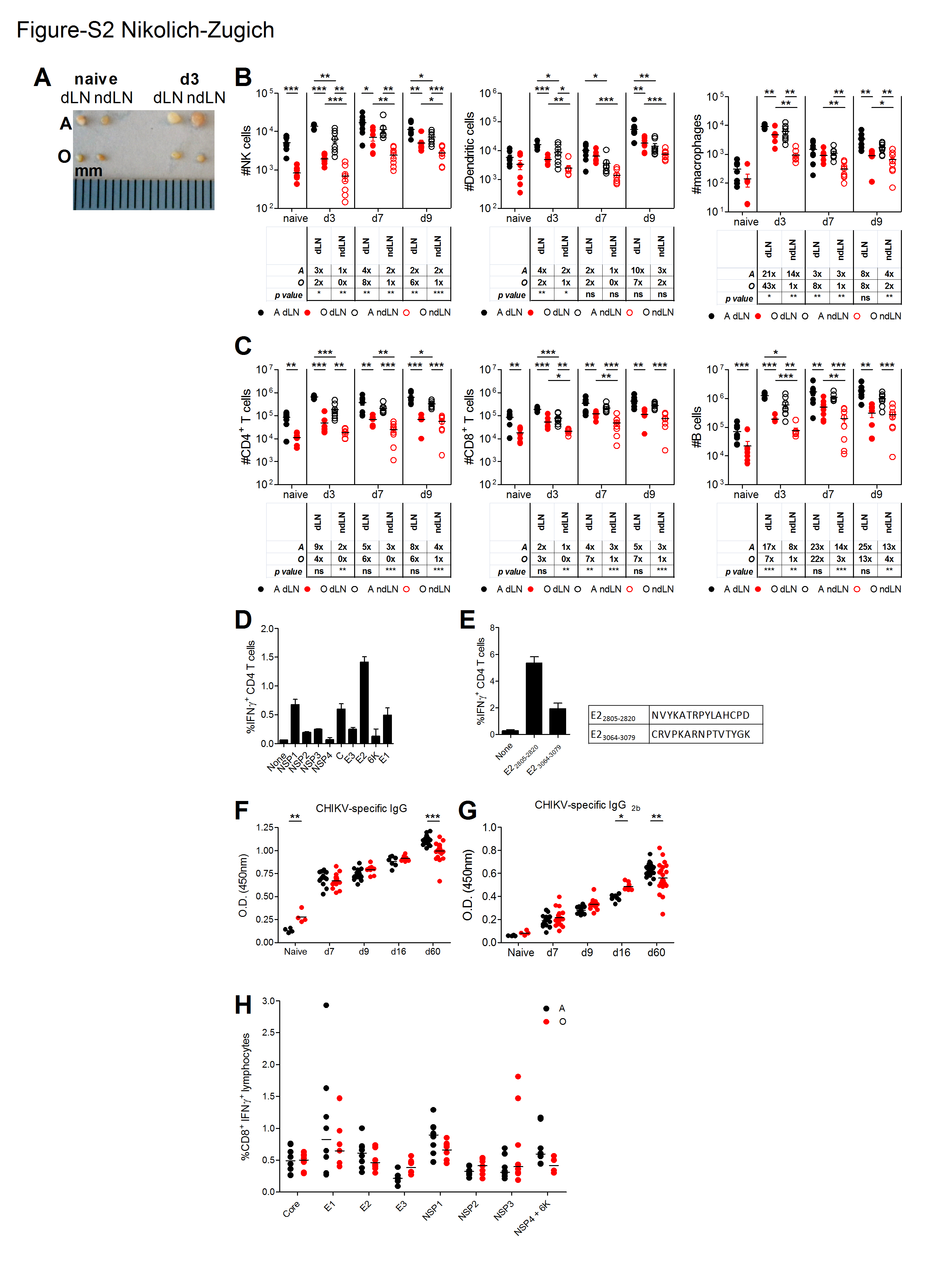

Supplement: S2 Fig — (A) Visual disparity between popliteal LNs collected from either naïve or CHIKV-infected A and O mice at day 3 post-infection. The LN draining from the CHIKV-inoculated foot is indicated as dLN and from the non-inoculated foot as ndLN in all panels. (B-C) Absolute number of NK cells, dendritic cells (DCs), macrophages, CD4+ T cells, CD8+ T cells, and B cells as determined by FCM analysis. Table under graph indicates the average fold-increase from naïve for each age in either the dLN or ndLN (n = 6–8 per group). Horizontal lines indicate the median. Statistical significance determined by student’s t-test. (D) dLN stimulated with overlapping peptide pools for each region of CHIKV in the presence of protein transport inhibitor to determine frequency of IFNγ+ CD4+ T cells. (E) dLN stimulated with individual peptides from the E2 region of CHIKV to determine dominant epitope. (F) CHIKV-specific total IgG and (G) IgG2b in serum was determined by ELISA at the indicated day post-infection. Data are mean (n = 4–24 per group). (H) Splenocytes stimulated with overlapping peptide pools for each region of CHIKV in the presence of protein transport inhibitor to determine frequency of IFNγ+ CD8+ T cells. Statistical significance was determined by two-way ANOVA with Bonferroni post-test. *P< 0.05; **P< 0.01; ***P< 0.001. (TIF) [file ppat.1005891.s002.tif]

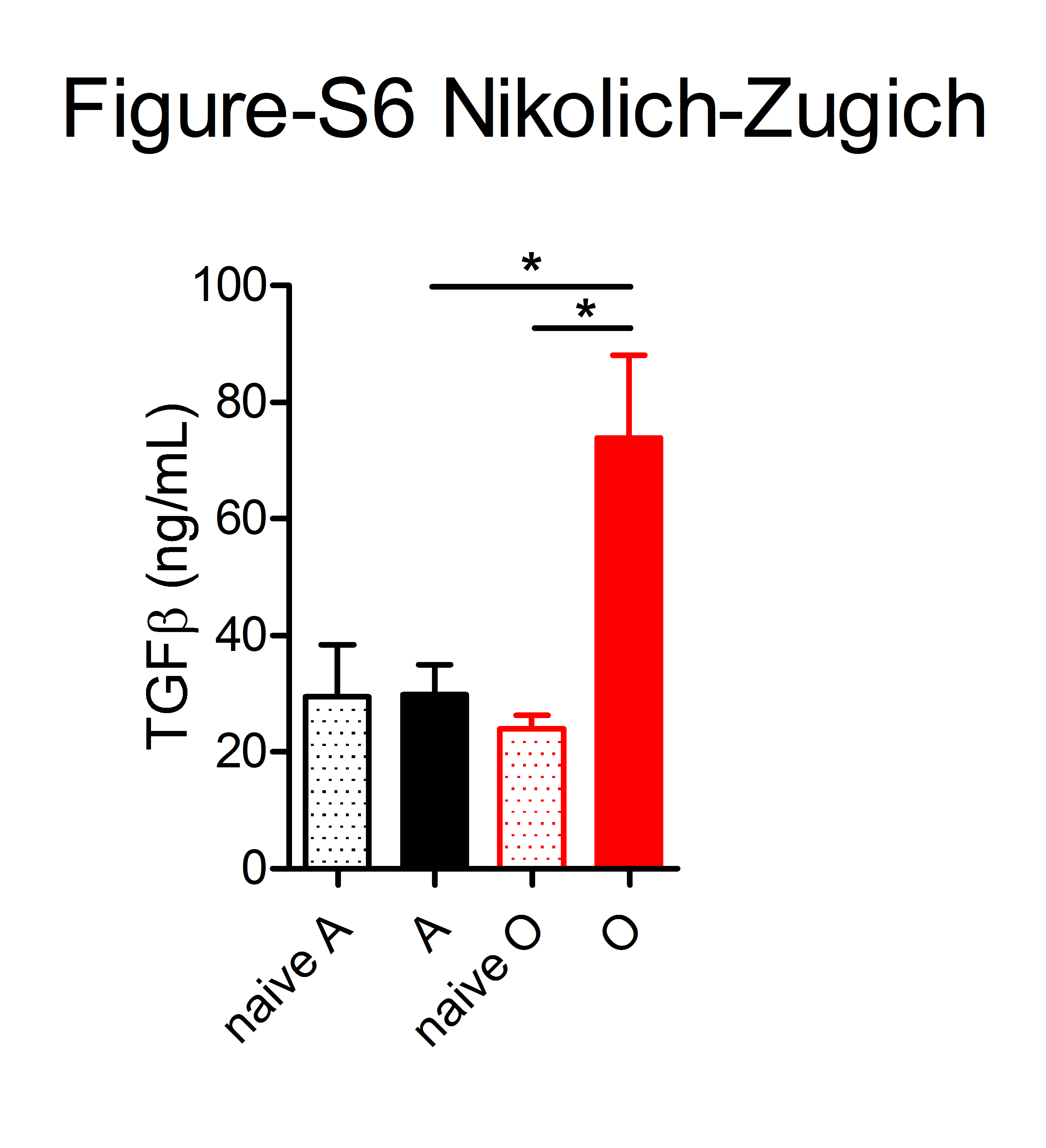

Supplement: S6 Fig — Serum was collected from A and O mice and assayed by ELISA for TGFβ concentration at day 10 post-infection. Data are mean + SEM (n = 7–8 naïve and 7–10 infected per age). Statistical significance was evaluated by unpaired student’s t-test. *P< 0.05. (TIF) [file ppat.1005891.s006.tif]
